# Supplementary material for: Diamide-based screening method for the isolation of improved oxidative stress tolerance phenotypes in Bacillus mutant libraries
Source: Microbiol Spectr. 2023 Oct 11;11(6):e01608-23. doi: 10.1128/spectrum.01608-23 (PMC10714788; doi:10.1128/spectrum.01608-23)
Supplement: Fig. S1 — Establishing the suitable diamide inhibition concentration for the parental strain DB430 ΔlipA for the microtiter plate kinetic growth assay. [file spectrum.01608-23-s0001.pdf]

Supplementary material

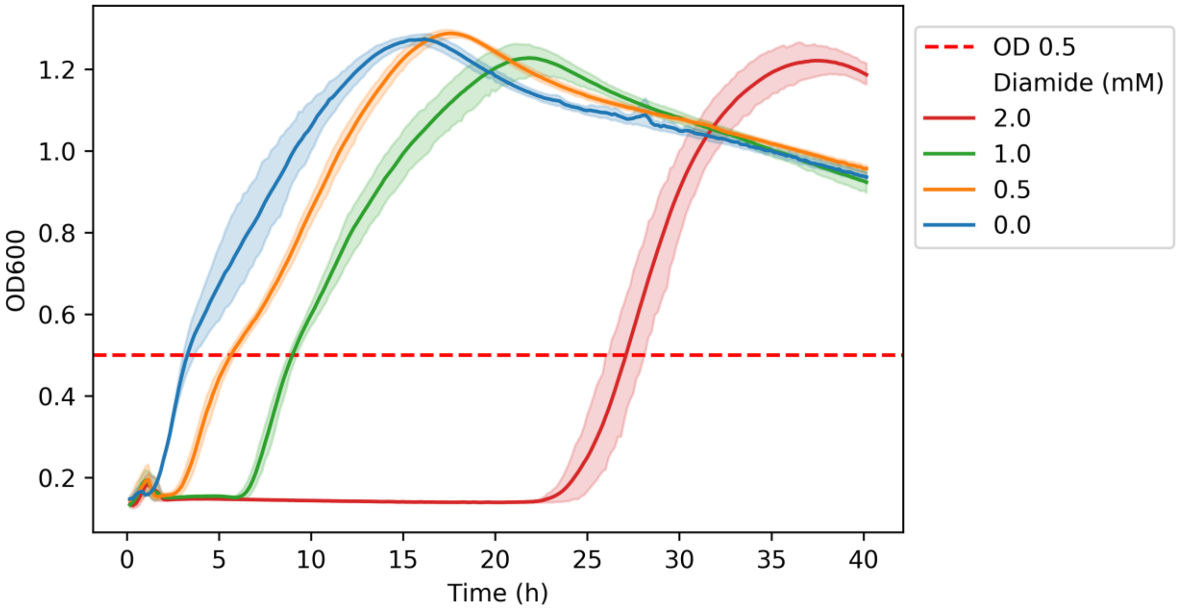

Figure S1: Establishing the suitable diamide inhibition concentration for the parental strain DB430 ΔlipA for the microtiter plate kinetic growth assay.
