# Supplementary material for: Diamide-based screening method for the isolation of improved oxidative stress tolerance phenotypes in Bacillus mutant libraries
Source: Microbiol Spectr. 2023 Oct 11;11(6):e01608-23. doi: 10.1128/spectrum.01608-23 (PMC10714788; doi:10.1128/spectrum.01608-23)
Supplement: Fig. S2 — Variation in growth inhibition times indicated by the time to reach an OD600 of 0.5 for the parental strain at different diamide concentrations. [file spectrum.01608-23-s0002.pdf]

Supplementary material

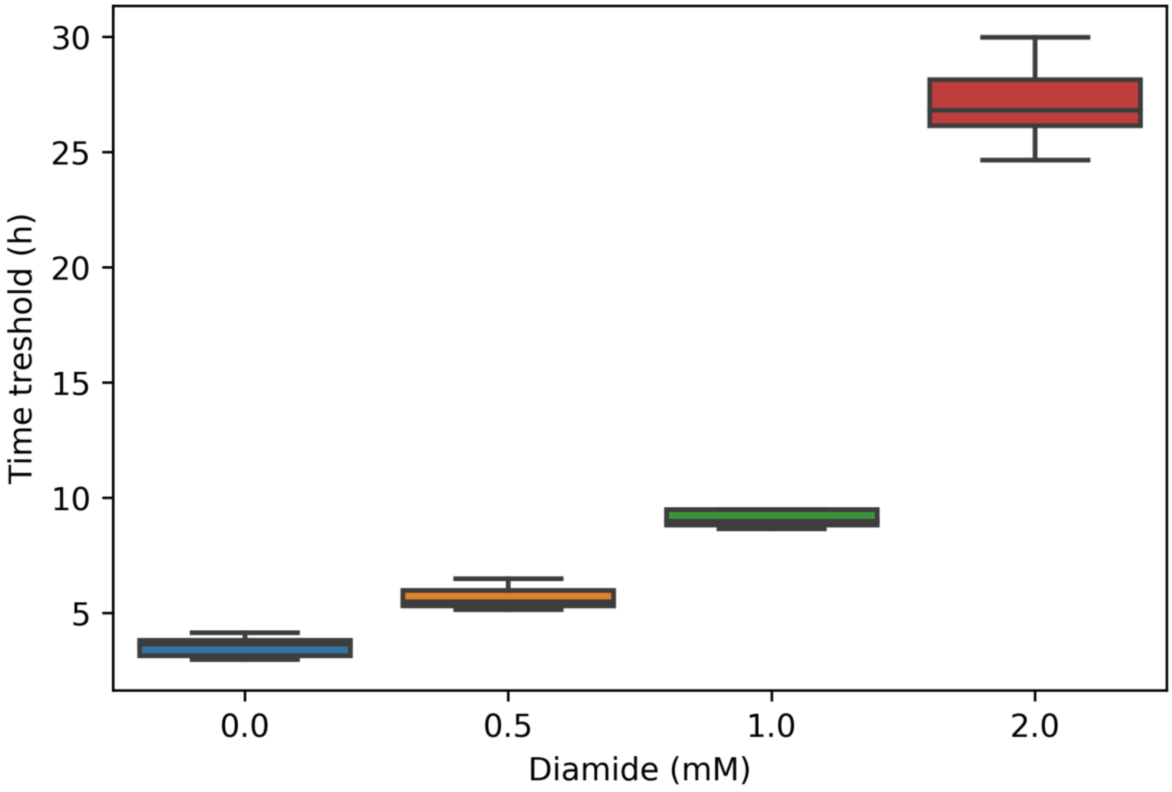

Figure S2: Variation in growth inhibition times indicated by the time to reach an OD_{600} of 0.5 for the parental strain at different diamide concentrations.
