# Supplementary material for: Diamide-based screening method for the isolation of improved oxidative stress tolerance phenotypes in Bacillus mutant libraries
Source: Microbiol Spectr. 2023 Oct 11;11(6):e01608-23. doi: 10.1128/spectrum.01608-23 (PMC10714788; doi:10.1128/spectrum.01608-23)
Supplement: Fig. S3 — Volcano plots comparing the abundance of proteins of each investigated strain at the different sampling points (0.5 h, 1 h, and 4 h) in the absence or presence of diamide, respectively. [file spectrum.01608-23-s0003.pdf]

Supplementary material

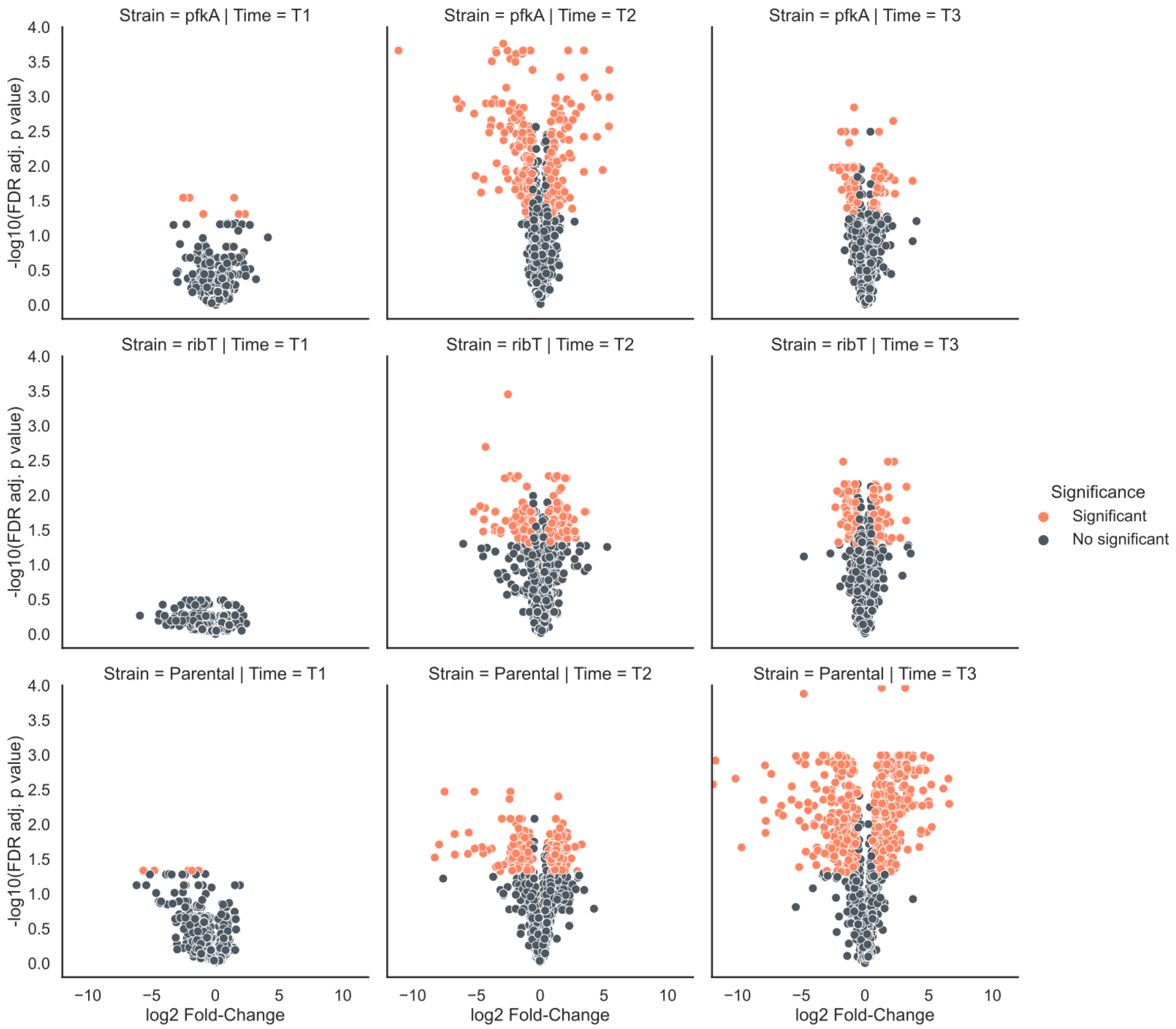

Figure S3: Volcano plots comparing the abundance of proteins of each investigated strain at the different sampling points (0.5 h, 1 h, and 4 h) in the absence or presence of diamide, respectively. Proteins whose abundance was significantly changed are indicated in orange.
