# Supplementary figures and images for: Diamide-based screening method for the isolation of improved oxidative stress tolerance phenotypes in Bacillus mutant libraries
Source: Microbiol Spectr. 2023 Oct 11;11(6):e01608-23. doi: 10.1128/spectrum.01608-23 (PMC10714788; doi:10.1128/spectrum.01608-23)

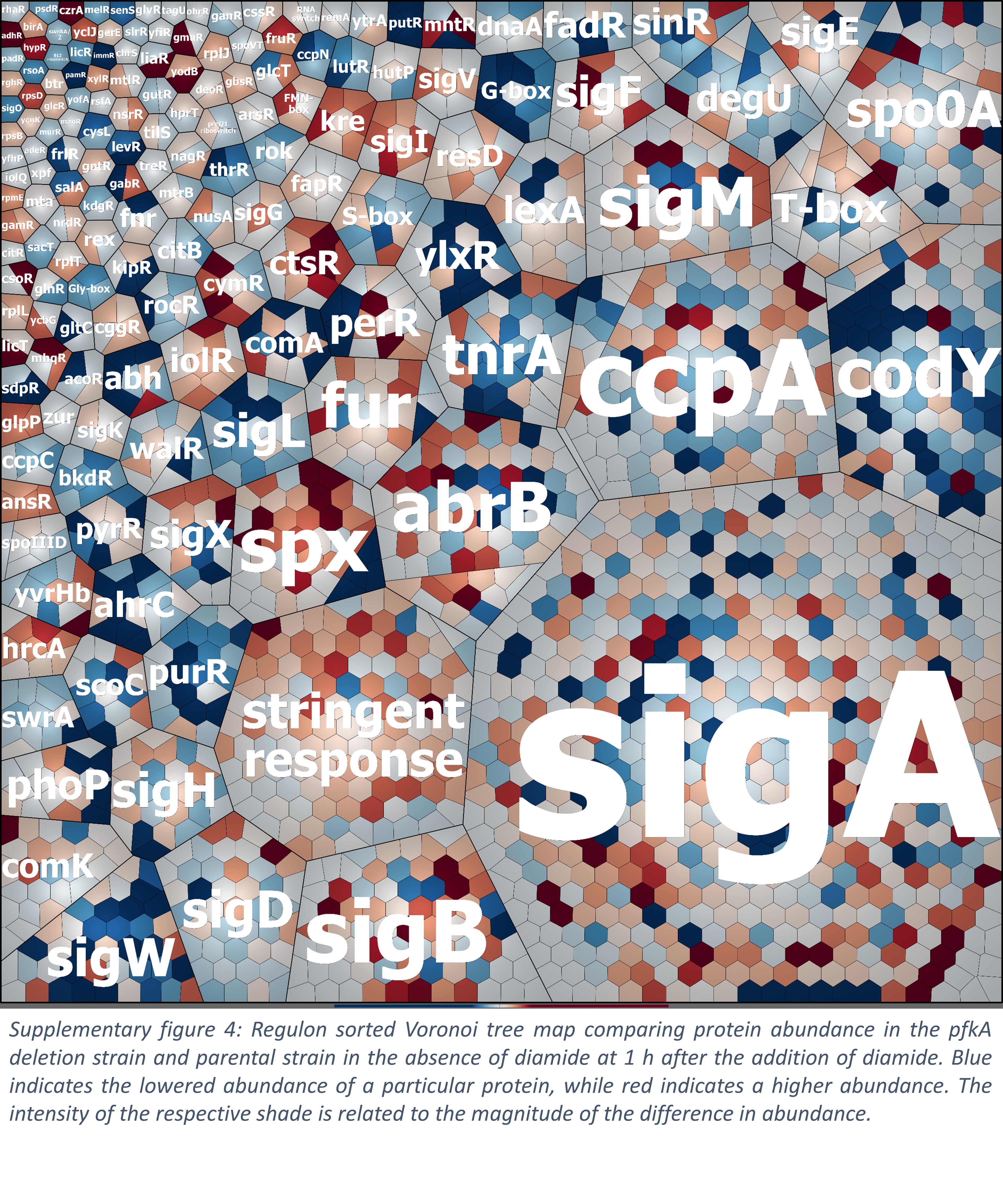

Supplement: Fig. S4 — Regulon sorted Voronoi tree map comparing protein abundance in the pfkA deletion strain and parental strain in the absence of diamide at 1 h after the addition of diamide. [file spectrum.01608-23-s0004.jpg]

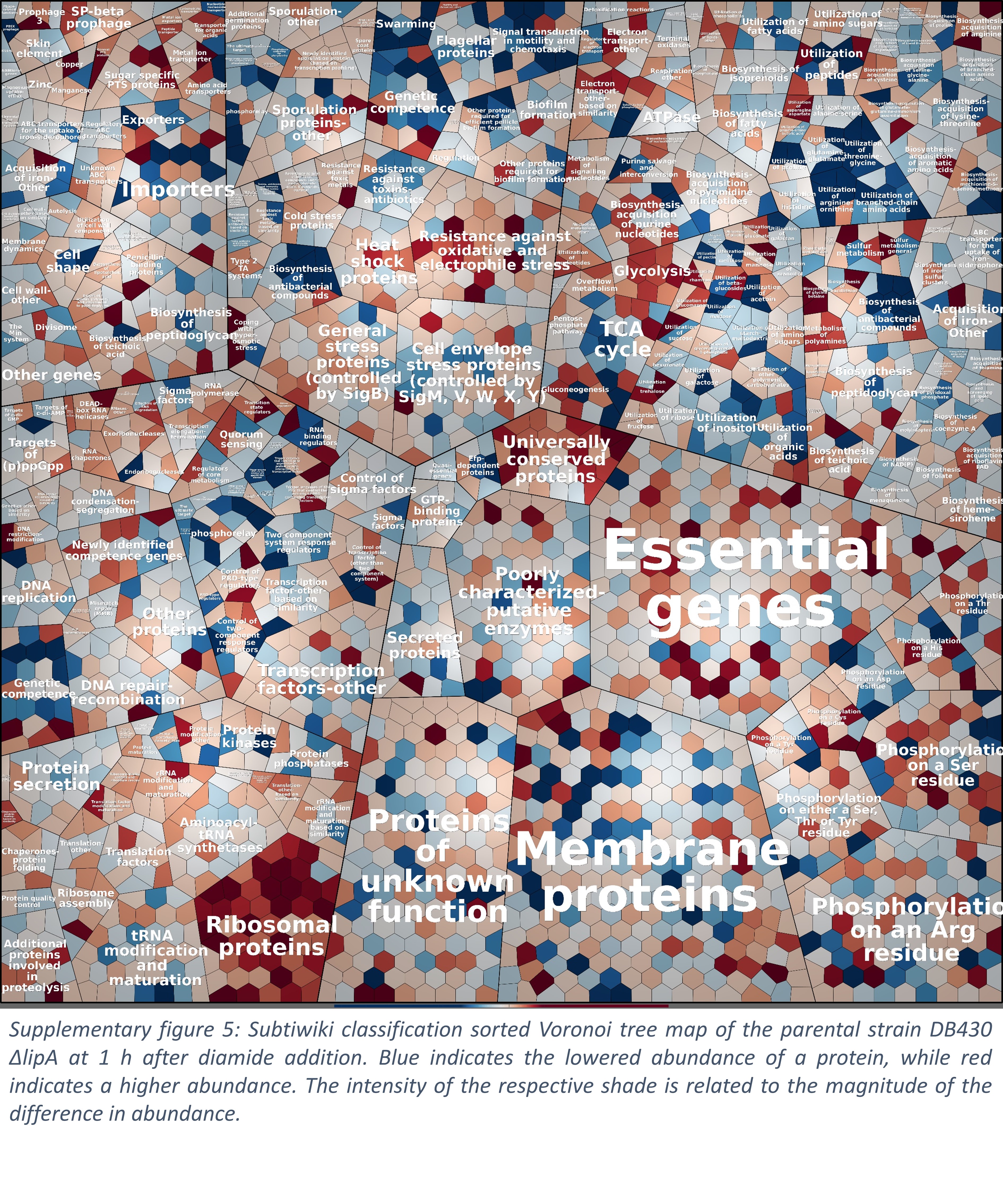

Supplement: Fig. S5 — Subtiwiki classification sorted Voronoi tree map of the parental strain DB430 ΔlipA at 1 h after diamide addition. [file spectrum.01608-23-s0005.jpg]

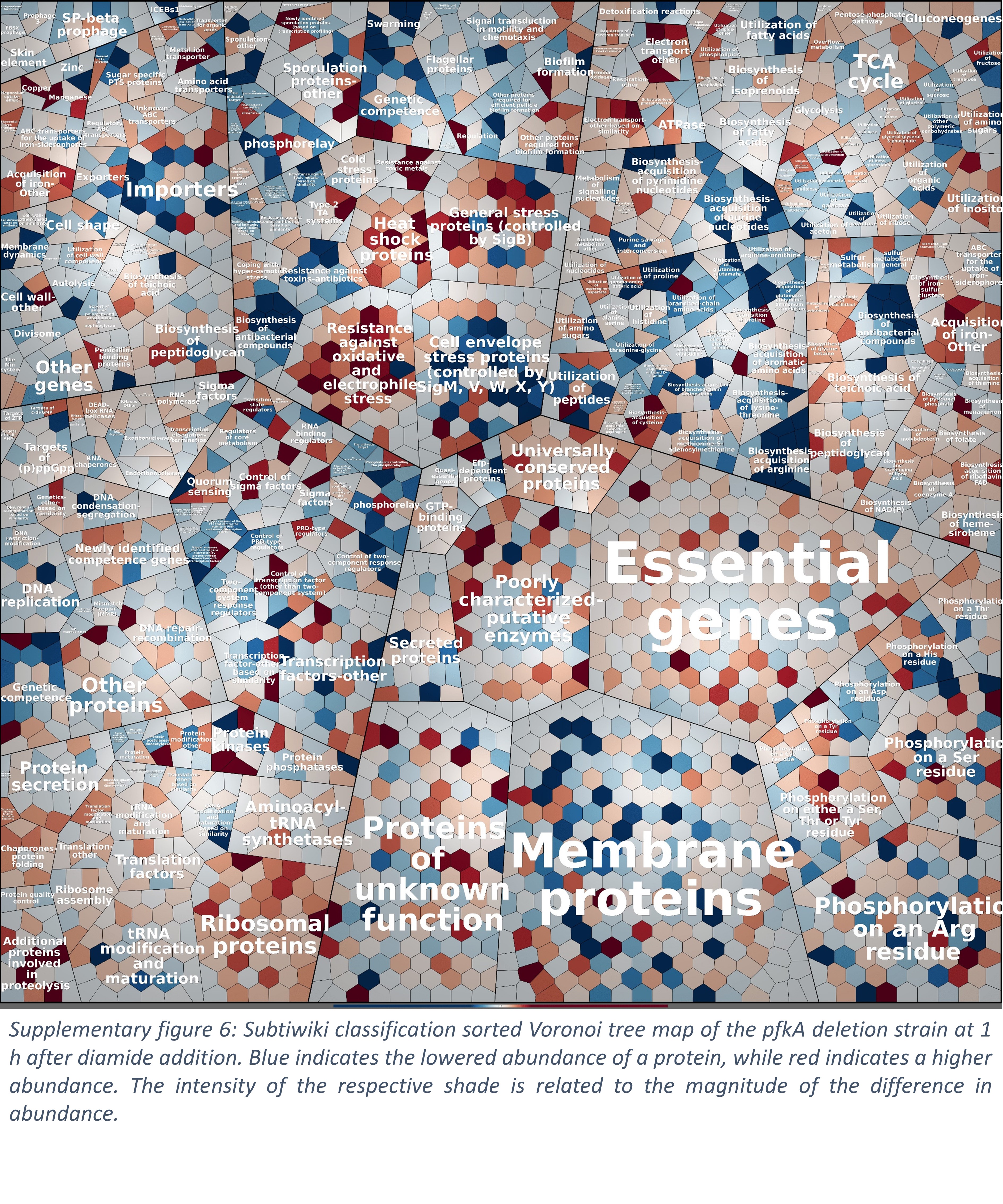

Supplement: Fig. S6 — Subtiwiki classification sorted Voronoi tree map of the pfkA deletion strain at 1 h after diamide addition. [file spectrum.01608-23-s0006.jpg]

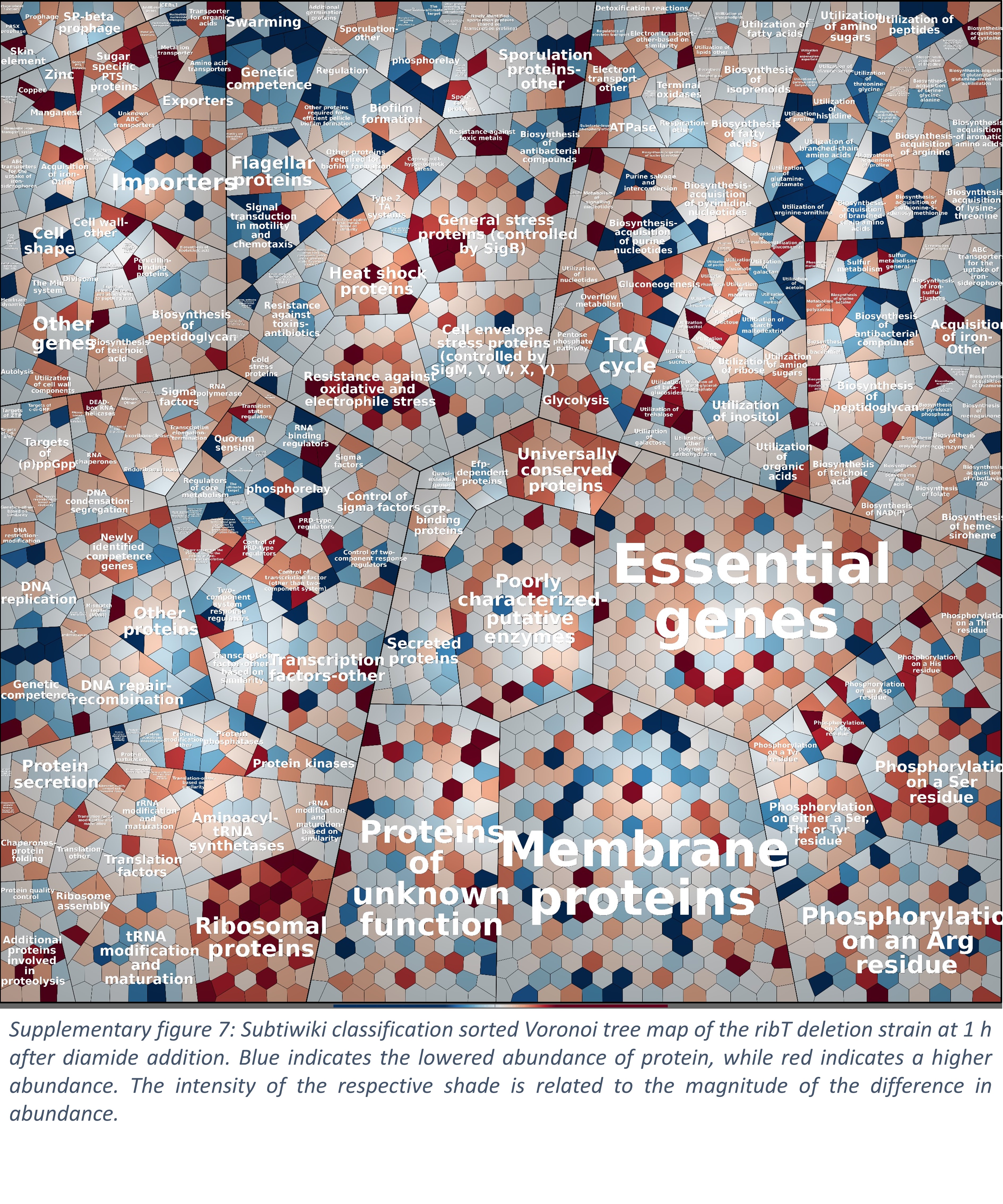

Supplement: Fig. S7 — Subtiwiki classification sorted Voronoi tree map of the ribT deletion strain at 1 h after diamide addition. [file spectrum.01608-23-s0007.jpg]
