# Supplementary material for: Diamide-based screening method for the isolation of improved oxidative stress tolerance phenotypes in Bacillus mutant libraries
Source: Microbiol Spectr. 2023 Oct 11;11(6):e01608-23. doi: 10.1128/spectrum.01608-23 (PMC10714788; doi:10.1128/spectrum.01608-23)
Supplement: Tables S1 and S2 — Primer list and summary of transposition events and CFU counts to estimate the total number of transposon mutants screened during the study. [file spectrum.01608-23-s0008.docx]

# Supplementary material

**Table S1: List of primers used during this study and their sequences.**

| Primer | Sequence |
| --- | --- |
| 2step_3 | ACAAGTGGTATGACATTGCC |
| 2step_4 | CGAAGAGGAACTTGTCTTTTCCC |
| 2step_seq_3 | AAGTTCGCTAGATAGGGGTCC |
| FAP_1 | GCGTTCAACTTTGGGAGAGACTGGC |
| pAL13_sso_4 | GCGTTCAACTTTGGGAGAG |
| SEC0227_rny_pKVM2_fw | GCTTCTAGAATTCGAGCTCCCGGGTAGCTATGAAAAGATGTTTACGCCAGGGAG |
| SEC0228_rny_pKVM2_rev | CAGATCTATCGATGCATGCCATGGTGTACGGTGTTCTTTTCGCAGCTTC |
| SEC0229_rny_rev | GCTTAGCGCATCACTACTTTCACCTCCTCTTGCTATG |
| SEC0230_rny_fw | AGAGGAGGTGAAAGTAGTGATGCGCTAAGCATC |
| SEC0231_pfkA_pKVM2_fw | GCTTCTAGAATTCGAGCTCCCGGGTATTCTGTTATTTCTCCGGAAGG |
| SEC0232_pfkA_pKVM2_rev | CAGATCTATCGATGCATGCCATGGTTTGAGTGTTCCGTTGTTTAATAC |
| SEC0233_pfkA_rev | GCCTTCAGCTGTACATCTCCATTCACCTCAGCAACATATATG |
| SEC0234_pfkA_fw | TGAGGTGAATGGAGATGTACAGCTGAAGGCTGAAG |
| SEC0235_cysE_pKVM2_fw | GCTTCTAGAATTCGAGCTCCCGGGTTGAAGAGCTTGAGGAGTTC |
| SEC0236_cysE_pKVM2_rev | CAGATCTATCGATGCATGCCATGGTTAGTTCATCGATTGATTGCTGAGAAAG |
| SEC0237_cysE_rev | TCATTTTTGATTGATATGCTTCCCCCCGTTTCT |
| SEC0238_cysE_fw | AACGGGGGGAAGCATATCAATCAAAAATGACAATCAC |
| SEC0239_pchR_pKVM2_fw | GCTTCTAGAATTCGAGCTCCCGGGTTATTTCTCTGCAGTTTTCTGTTAATG |
| SEC0240_pchR_pKVM2_rev | CAGATCTATCGATGCATGCCATGGTTGCTCCGCCTATGAATCC |
| SEC0241_pchR_rev | ACACCGCCTTTTTGTAGGCTACCTTCTTTCTTAGGAATAATAG |
| SEC0242_pchR_fw | GAAAGAAGGTAGCCTACAAAAAGGCGGTGTAC |
| SEC0243_ypzk_pKVM2_fw | GCTTCTAGAATTCGAGCTCCCGGGTTACGAGCAAGCTGCTGAG |
| SEC0244_ypzk_pKVM2_rev | CAGATCTATCGATGCATGCCATGGTTCATGCGACACGGGATTATTTTG |
| SEC0245_ypzk_rev | GATCACAGCCTCTGCAAAAACCCCTCTATATCCAAAATG |
| SEC0246_ypzk_fw | TATAGAGGGGTTTTTGCAGAGGCTGTGATCAGTC |
| SEC0247_bshC_pKVM2_fw | GCTTCTAGAATTCGAGCTCCCGGGTGTTTCAGCATAACCATTC |
| SEC0248_bshC_pKVM2_rev | CAGATCTATCGATGCATGCCATGGTTTCTGTGTATTGTTCCCAAATTAC |
| SEC0249_bshC_rev | CTTTAAAACTTTATTCTTCTAGAACTTCCTTTCTCCAATAAATAG |
| SEC0250_bshC_fw | AGGAAGTTCTAGAAGAATAAAGTTTTAAAGAACCCTGACTAG |
| pKVM_hsdR_test fw | ACGGTTCGATCTTGCTCCAACTG |
| pKVM_hsdR_test rev | CTACCGGTGAACCTGTTTGC |
| SEC0257_rny_5_fw | AGTAAGCGGTGAGCCCTATG |
| SEC0258_pfkA_5_fw | AGCGCTTCGTCTGATGAAAC |
| SEC0259_cysE_5_fw | AACTGACGCTTCCGCATTTG |
| SEC0260_pchR_5_fw | GGGCTCTTTCTGCAAATCTC |
| SEC0261_yzpk_5_fw | TCGGTGTCCGGAATATGAAG |
| SEC0262_bshC_5_fw | TGACAGTGAAGCAGCATCAG |

**Table S2: summary of transposition events and CFU counts to estimate the total number of transposon mutants screened during the study**

| Event | Plates | Volume plated (µL) | CFU counted 1 | CFU counted 2 | CFU counted 3 | Dilution | CFU/mL | amount viable screened |
| --- | --- | --- | --- | --- | --- | --- | --- | --- |
| TMA1 | 20 | 100 | 256 | 269 | 206 | 1.00E-02 | 2.44E+05 | 4.87E+05 |
| TMA2 | 20 | 100 | 10 | 9 | 9 | 1.00E-03 | 9.33E+04 | 1.87E+05 |
| TMA3 | 27 | 100 | 10 | 9 | 4 | 1.00E-03 | 7.67E+04 | 2.07E+05 |
| TMA4 | 30 | 100 | 15 | 18 | 16 | 1.00E-03 | 1.63E+05 | 4.90E+05 |
| Sum |  |  |  |  |  |  |  | **1.37E+06** |
